# VirusMapper: open-source nanoscale mapping of viral architecture through super-resolution microscopy

**DOI:** 10.1038/srep29132

**Published:** 2016-07-04

**Authors:** Robert D. M. Gray, Corina Beerli, Pedro Matos Pereira, Kathrin Maria Scherer, Jerzy Samolej, Christopher Karl Ernst Bleck, Jason Mercer, Ricardo Henriques

**Affiliations:** 1 MRC-Laboratory for Molecular Cell Biology. University College London, Gower Street, London, WC1E 6BT, United Kingdom; 2 Centre for Mathematics and Physics in Life Sciences and Experimental Biology (CoMPLEX), University College London, Gower Street, London, WC1E 6BT, United Kingdom; 3 Department of Cell and Developmental Biology, University College London, Gower Street, London, WC1E 6BT, United Kingdom; 4 Biozentrum, University of Basel, Klingelbergstrasse 50/70, CH-4056 Basel, Switzerland

## Abstract

The nanoscale molecular assembly of mammalian viruses during their infectious life cycle remains poorly understood. Their small dimensions, generally bellow the 300nm diffraction limit of light microscopes, has limited most imaging studies to electron microscopy. The recent development of super-resolution (SR) light microscopy now allows the visualisation of viral structures at resolutions of tens of nanometers. In addition, these techniques provide the added benefit of molecular specific labelling and the capacity to investigate viral structural dynamics using live-cell microscopy. However, there is a lack of robust analytical tools that allow for precise mapping of viral structure within the setting of infection. Here we present an open-source analytical framework that combines super-resolution imaging and naïve single-particle analysis to generate unbiased molecular models. This tool, VirusMapper, is a high-throughput, user-friendly, ImageJ-based software package allowing for automatic statistical mapping of conserved multi-molecular structures, such as viral substructures or intact viruses. We demonstrate the usability of VirusMapper by applying it to SIM and STED images of vaccinia virus in isolation and when engaged with host cells. VirusMapper allows for the generation of accurate, high-content, molecular specific virion models and detection of nanoscale changes in viral architecture.

Probing viral structure often provides novel insight into the structure-function relationship of protein localization within viral particles^1^,^2^. This information is key to understanding viral organization, and ultimately the mechanisms of virus metastability that dictate virus assembly and disassembly in host cells^3^. Due to their size the methods of choice for investigating the nanoarchitecture of viruses are electron microscopy (EM) and electron tomography (ET). Taking advantage of the stability of cell free viruses, single particle analysis (SPA) is often employed in EM and ET to achieve the resolution required to study viral architecture. Nonetheless, limitations with regard to combining these techniques with molecular specific labelling make it difficult to interrogate the location and identity of specific molecules within virus particles in a quick and reliable way. Super-resolution (SR) microscopy is a relatively new technique that provides the capacity to resolve molecular assemblies at scales considerably lower than the diffraction limit (~300 nm), while simultaneously allowing for the visualization of specific proteins within complex nanostructures. The recent application of SPA to SR microscopy data sets has proven particularly powerful for precision mapping of individual proteins within nanoscale molecular assemblies, including viruses^4^,^5^,^6^,^7^. However, the promise of combining SPA and SR microscopy is challenged by the lack of freely available easy-to-use compatible software packages. Here we describe an ImageJ and Fiji-compatible, high-throughput SPA framework that allows for automated detection, segmentation, alignment, classification and averaging of thousands of individual viruses for the generation of SR models of virus nanoarchitecture (Supporting Information Software). We have demonstrated the method with structured illumination microscopy (SIM) and stimulated emission depletion (STED) microscopy and the method is also expected to work for high quality single-molecule localization microscopy (SMLM) data, as long as the labelling and localisation density is high enough. Caution is advised for sparsely labelled data where our alignment method may no longer function correctly. VirusMapper is an open-source, user-friendly software that provides researchers with a powerful tool to create accurate, detailed virus models thereby opening new doors to further our understanding of virus structure-function relationships.

## Results

### VirusMapper workflow for model-based analysis of virus architecture

**Figure 1:**
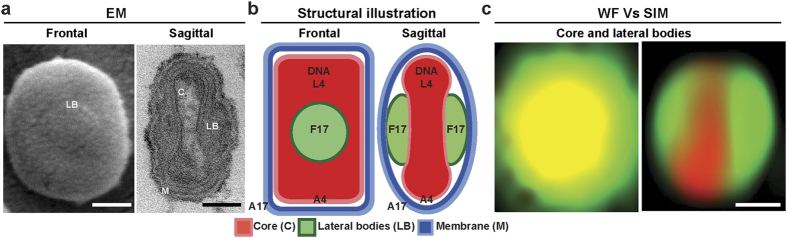
VACV structural organization. (**a**) Scanning (left) and transmission (right) EM of a VACV mature virion seen in frontal view and sagittal cross-section respectively. (**b**) Structural illustrations of MVs in sagittal cross-section and frontal views. MVs are composed of three major structural elements: a dumbbell shaped core (C; red), two lateral bodies (LB; green), and the viral membrane (M; blue), encompassing these elements. Sub-viral locations of the VACV proteins and components referred to in this report are illustrated. (**c**) Wide-field (left) vs structured illumination microscopy (right) images of a single VACV virion. Scale bars = 100 nm.

To determine the structural model generation ability of VirusMapper we applied it to the prototypic poxvirus, vaccinia virus (VACV)^8^. The major infectious form of VACV, mature virions (MVs), are brick shaped particles measuring 360 × 270 × 250 nm^9^. The MVs are composed of ~80 different proteins^8^,^10^ divided between 3 viral substructures discernable by EM: a dumbbell shaped core that contains the DNA genome, two proteinaceous structures termed lateral bodies (LBs) that sit within the core concavities, and a single lipid bilayer membrane that encompasses these structures (Fig. 1a,b). EM and ET have provided information about the general structure of VACV^11^. Biochemical fractionation studies have been used to differentiate viral membrane from core proteins^12^,^13^, and immuno-EM has provided the localization of a small subset of proteins within virions^14^,^15^,^16^.

The well-known structural organization, and the ability to specifically visualize multiple virion proteins or components simultaneously makes VACV MVs an excellent system to showcase the potential of VirusMapper. To generate multi-color SR images, purified viruses were either bound to coverslips or exposed to cells and imaged by either SIM or STED.

The combination of VirusMapper and super-resolution provides several advantages: i) for VACV it provides the capacity to separate virion substructures (Fig. 1c); ii) it allows the use of a combination of labelling strategies; iii) easy acquisition of multi-color SR images; and iv) hundreds to thousands of viruses can be imaged within a single field of view with minimal optical distortion.

**Figure 2:**
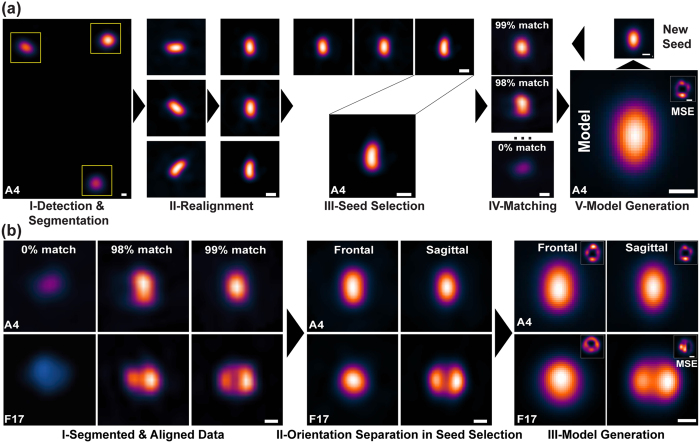
Information extraction using VirusMapper: Segmentation, Seed Selection, and Model Generation. (**a**) VirusMapper workflow: *Detection & Segmentation*: a particle detection algorithm detects and realigns (Realignment) viral particles based on their shape and symmetry (VACV A4 pictured). *Seed Selection*: from the aligned particles a subset of representative viral images is selected based on user-defined characteristics (*a priori* knowledge of core shape). *Model Generation*: the aligned particles are matched against the seed (Matching) and normalized by cross-correlation similarity to obtain the model MSE and concomitantly to iterate the process until the smallest MSE is obtained. The resulting models can also serve as new seeds for further model improvement. (**b**) Multi-component model generation. The process illustrated in (A) can be repeated to generate multi-component models of single virions. For this, more than one seed selection criteria is applied to a single reference channel (VACV A4 frontal and sagittal pictured). Additional virion components (VACV F17 pictured) are aligned to the reference for the generation of virion orientation-based (frontal and sagittal) models of various viral components. Scale bars = 100 nm.

With SIM imaging we acquired images of a recombinant virus that packages a mCherry-tagged version of the outer core protein A4. We reasoned that the virus core was the ideal structural element to initiate mapping as its near ellipsoid shape offered the possibility of determining particle orientation. After acquisition, images were data mined by VirusMapper in three main stages: segmentation, seed selection, and model generation (Fig. 2 and Supplementary Fig. 1). For segmentation a particle detection algorithm automatically finds and realigns viral particles based on the oblong appearance of A4 (Fig. 2a, I). From these, an image sequence of individual virions oriented with their long axis in the vertical position is generated (Fig. 2a, II). Next, a small subset of representative virus core images is selected based on *a priori* knowledge and highlighted as seeds for template matching (Fig. 2a, III). Following seed selection, each individual viral particle detected and segmented from the SIM images is registered in its rotation and coordinates against the given seed and sorted by normalized cross-correlation similarity (Fig. 2a, IV and Supplementary Note 1). For model generation, the registered core images are averaged using the normalized cross-correlation similarity factor as weights, consequently a Mean Square Error (MSE) of the model against individual elements is also calculated. The process of alignment and template matching is iterated using each newly generated model as a seed, minimizing the MSE of the model (Fig. 2a, V and IV).

### Multi-seed mapping for virus orientation assessment

Having established a model of the viral core by mapping A4, we next labelled multiple virus proteins in combination to map their locations within viral substructures. We collected SIM images of recombinant virions that package fluorescently tagged versions of the inner core protein L4 and the major LB constituent F17. As before, collected images were processed by VirusMapper. Analysis is initiated with virion segmentation and realignment for both channels in parallel (Fig. 2b, I). For multi-color model generation in VirusMapper a reference channel is selected, in this instance A4, on which the registration estimation is based and then applied to all channels (Fig. 2b, III). By selecting multiple A4 seeds to probe the dataset for F17 mapping, two models of F17 position emerged: in the first F17 was confined to a single circular structure centred on A4, in the second two oblong F17 structures horizontally flank the A4 core (Fig. 2b, III). Illustrating the power of VirusMapper for unbiased model generation, using multiple seed selection we obtained protein localization models that accurately represent the frontal and sagittal views of VACV particles as phenotypically defined by EM (Fig. 1a and ref. 9).

### High-precision six-component model of VACV nanoarchitecture

These results suggested that it is possible to map a large number of viral proteins or components using the same viral feature as a reference. To this end we acquired multi-color SIM images of various viral components in combination with the core proteins A4 or L4 as reference channels for mapping. To visualize membrane proteins, virions were subjected to immunofluorescence directed against the membrane protein A17. The virus lipid membrane was visualized using the lipid dye CellMask. As in Fig. 1, to visualize the virus core and LBs, recombinant viruses expressing fluorescently tagged versions of the outer core protein A4, the inner core protein L4, or the LB constituent F17 were used. Finally, the DNA dye TO-PRO-3 was used for visualization of viral genomes.

**Figure 3:**
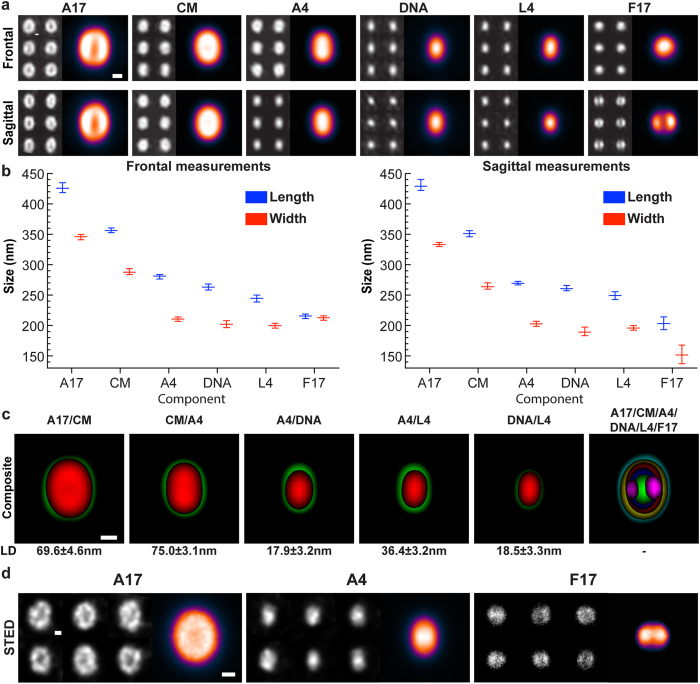
Mapping of VACV components in multiple SR modalities. (**a**) Frontal and sagittal models of six virion components generated using VirusMapper including membrane protein A17, the viral lipid bilayer (Cell Mask; CM), viral outer core protein A4, the viral DNA, the inner core protein L4, and the LB component F17. Scale bars = 100 nm. Representative individual particles (left; grey) and resulting models (right; color). (**b**) Length and width measurements of each virion model. Bars represent the range of the cross-validation distributions for each. (**c)** Models of vaccinia virus nanoarchitecture. Composite images of size descending frontal models and their precision (LD; length difference) as well as a composite sagittal model of all mapped virion components: A17 (green)/CM(red), CM(green)/A4(red), A4(green)/DNA(red), A4(green)/L4(red), DNA(green)/L4(red) and a composite of all sagittal models A17(cyan)/ CM(yellow)/ A4(red)/ DNA(blue)/ L4(green)/ F17(magenta). Scale bar = 100 nm. **(d)** STED models of the three major subviral structures. Membrane protein A17, core protein A4 (both frontal orientation) and lateral body protein F17 (sagittal orientation). Representative individual particles (left; grey) and resulting models (right; color). Scale bars = 100 nm.

Using VirusMapper we generated 2D, SR frontal and sagittal models of each virion component complete with their MSE (Fig. 3a and Supplementary Fig. 2). Measurements of each component in both frontal and sagittal views showed that the length and width of those imaged decreased as follows: viral membrane protein A17, the viral membrane, outer core protein A4, viral DNA, the inner core protein L4, and the LB protein F17 (Fig. 3b and Supplementary Table 1).

Next, to access the accuracy of this approach, we compared the measurements of our model VACV with those obtained by cryo-ET and atomic force microscopy (AFM)^9^,^17^. Consistent with the cryo-ET [360(l) × 270(w) × 250(h) nm] and AFM [300–360(l) × 240–280(w) × 240–290(h) nm] measurements of intact VACV, our model VACV has an average size of 356 (l) × 288 (w) × 264(h) nm as determined by CellMask labelling (Fig. 3b and Supplementary Table 1). The core length of our model (281 ± 3 nm) was also consistent with that previously reported for VACV by ET (275 ± 18 nm)^18^. When measurements were made of the VACV model generated using images of the viral membrane protein A17, we noted a ~60 nm increase in virion size (420 × 337 × 314 nm) (Fig. 3b and Supplementary Table 1). As A17 was visualized by immunofluorescence this increase suffers from the inherent error contribution of using primary and secondary antibodies in SR microscopy^19^. We also noted that although the viral genome and DNA binding protein L4 largely overlap, the DNA extended beyond the L4 signal towards the periphery of the core (Fig. 3b and Supplementary Table 1). This observation is in agreement with cryo-ET data showing the viral DNA in close association with the core wall^9^,^18^.

That differences in the size of virion components residing in the core substructure (A4, DNA and L4) were seen demonstrates that the models generated by VirusMapper are of high precision. Composite images of closely apposed virion components, (A17 and the viral membrane, viral membrane and outer core protein A4, A4 and inner core protein L4, A4 and the viral DNA, L4 and the viral DNA) as well as the precision to which they could be mapped were generated (Fig. 3c). We found that the individual components could be mapped against each other within a distance of 19.7–69.6 nm with a precision of 3.1–4.6 nm as the standard deviation of the cross validation distribution.

### VirusMapper can be applied to multiple super-resolution modalities

To investigate the applicability of VirusMapper to super-resolution techniques other than SIM we imaged the VACV membrane, core and LBs using stimulated emission depletion (STED) microscopy. To visualize the viral membrane, the viral membrane protein A17 was stained with Alexa488 labelled antibodies. For visualization of the viral core and LBs, recombinant viruses with EGFP-tagged versions of the core protein A4 and the LB protein F17 were used. To aid particle detection and alignment, A17 and F17 were simultaneously imaged with mCherry-tagged A4.

After STED imaging, VirusMapper was used to generate models of these three viral components (Fig. 3d). Although we were unable to match the resolution of the SIM models due to considerable fluorophore photobleaching at high depletion beam illumination, VirusMapper was successfully implemented to create accurate models of the three major viral components using images generated by STED microscopy. These results demonstrate that VirusMapper can be applied to images acquired using various super-resolution methods.

### VirusMapper can accurately resolve nanoscale changes in virus architecture

**Figure 4:**
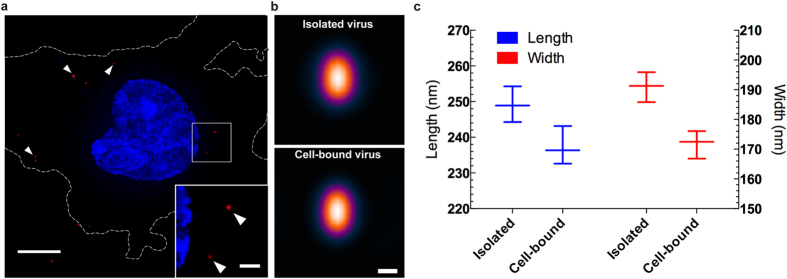
Detection of nanoscale changes in virion architecture using VirusMapper. **(a)** Purified VACV mCherry-L4 virions were bound to cells for 60 minutes at 25 °C and samples were fixed prior to staining with Hoechst to visualize nuclei. Samples were imaged by SIM and Z-stacks collected over the full cell height. The maximum intensity projection of a representative cell with bound virions (arrow heads and inset) is shown. Scale bar 5 μm, inset scale bar 1 μm **(b)** VirusMapper generated models of L4 protein in isolated and cell-bound viruses. Scale bar 100 nm. **(c)** Length and width measurements of L4 virion models in (b). Note different scales for length and width measurements.

That extracellular viruses are highly stable structures dictates that their structure encodes inherent metastability to allow for efficient genome delivery and subsequent progeny virion assembly within the same host cells^3^,^20^. These structural changes can occur as early as virus-cell binding, and in these cases are so minute that they have only been detected in a limited set of viruses using EM and ET^2^,^18^. Using whole cell cryo-ET, it was suggested that upon cell contact VACV cores undergo a shape change that correlates with genome de-condensation^18^. However, this was concluded from a single VACV particle in the absence of molecular specific labelling. Therefore, we set out to elucidate the nature of these changes by applying VirusMapper to SIM images of cell-bound fluorescent VACV virions. For this mCherry-L4 VACV was bound to HeLa cells at 25 °C for 1 hour. At this temperature virions can attach to the cell surface but cannot be internalized^21^,^22^. After binding, cells were fixed and imaged by SIM. To assure all cell bound virions were visualized Z-stacks were collected through the height of the cells and converted to maximum intensity projections (Fig. 4a). The images were analysed by VirusMapper and models of L4 protein generated by analysis of approximately one thousand virions (Fig. 4b). The resulting models indicated that L4 within virions bound to the cell surface had condensed toward the center of the virion core. Measurement indicated that the position of L4 within virions had shifted away from the core wall by 20 nm in width and 13 nm in length relative to its position in isolated virions (Fig. 4c). As a viral DNA binding protein, the observed contraction of L4 is consistent with the release of the viral DNA from the core wall upon cell binding. These results illustrate that VirusMapper can be used to investigate minute nanoscale changes in virus metastability in the context of host cells at high-throughput and with great precision.

## Discussion

VirusMapper enables the mapping of virus protein architecture by harnessing the power of SR microscopy and SPA. We were able to generate high precision multi-component 2D models of viral protein and substructure localization, and investigate nanoscale changes in viral protein architecture upon cell binding, each of which would have been impossible using conventional light microscopy, EM or ET. EM and ET have been valuable tools to determine the overall structural organization of viruses, including VACV^9^,^11^, and SR has already offered a look into the structural organization of some viruses^4^. When coupled with correlative EM technologies and applied to complex dynamic intracellular processes, such as virus uncoating or assembly, VirusMapper has the potential to facilitate understanding of the molecular details of virus structural metastability with high precision and accuracy.

There are many software packages available for single particle analysis of data generated using cryo-EM^23^. However these require parameters not easily translated to light microscopy and are highly specialised for producing 3D reconstructions of protein structure. They are therefore not readily applicable to fluorescence imaging. The same averaging principles have been successfully applied to fluorescence^1^,^2^,^3^,^4^, but are not available as an easy-to-use, freely available package for the popular ImageJ and Fiji image analysis software.

By providing a fast, user-friendly ImageJ and Fiji^23^ based analytical framework for the naïve averaging and model extraction of SR images, we equip researchers with the possibility to map an unlimited number of proteins and components within viruses and other nanoscale macromolecular complexes at high precision on robust 2D and 3D models. Thus VirusMapper provides the opportunity to discover novel virus structures and uncover new structure-function relationships within virion protein architecture, which in turn may facilitate the development of effective antiviral agents.

## Methods

### Reagents

#### Cell Lines

BSC-40 cells were cultivated in Dulbecco’s modified Eagle’s medium (DMEM) supplemented with 10% heat-inactivated fetal bovine serum (FBS), 2 mM Glutamax, 100 units/mL penicillin and 100 *μ*g/mL streptomycin, 100 *μ*M nonessential amino acids and 1 mM sodium pyruvate.

#### Viruses

Recombinant VACV were based on VACV strain Western Reserve (WR). WR mCherry-A4 and WR GFP-A4 were previously described as WR mCherry-A5 and WR GFP-A5 24. WR L4-mCherry EGFP-F17 was previously described as WR EGFP-F17 VP8-mCherry^16^. MVs were produced in BSC-40 cells, purified from cytoplasmic lysates and banded on a sucrose gradient, as previously described^25^.

#### Antibodies and Dyes

Rabbit polyclonal antibody anti-A17 (p21 N-terminal)^15^ was a kind gift of Jacomine Krijnse-Locker (Institut Pasteur, Paris, France). AlexaFluor 488 and AlexaFluor 594 coupled goat anti-rabbit secondary antibodies, the membrane stain CellMask^™^ Deep Red and Green Plasma membrane stains and the nucleic acid stain TO-PRO-3 Iodide were purchased from Life Technologies.

### Super-Resolution Microscopy Sample Preparation

High performance coverslips (18 × 18 mm 1.5 H, Zeiss) were washed as previously described^26^. Purified virus was diluted in 10 mM Tris pH 9, bound to the ultra-clean coverslips for 30 min and fixed with 4% Formaldehyde. To visualize the viral DNA, virus bound to the coverslip was incubated with 1 μM TO-PRO-3 Iodide for 1 hour. Samples were washed 3 times with PBS prior to mounting for imaging. To visualize the viral membrane, virus was blocked after fixation using 5% FBS in Phosphate Buffered Saline (PBS) for 30 min, incubated in 1% BSA in PBS with primary anti-A17 antibody, followed by (488 or 594) goat anti-rabbit fluorescent secondary antibodies for 1 hour each. Three PBS washes of 10 min each were performed between each staining step. For CellMask staining, 5 *μ*l of purified virus was incubated with 1 *μ*l CellMask for 20 min and washed twice with 500 *μ*l of PBS. The coverslips corresponding to the different stainings were mounted in Vecta Shield (Vector Labs).

### Super-resolution Structured Illumination Microscopy (SR-SIM)

SR-SIM imaging was performed using Plan-Apochromat 63 × /1.4 oil DIC M27 objective, in an Elyra PS.1 microscope (Zeiss). Images were acquired using 5 phase shifts and 3 grid rotations, with the 647 nm (32 μm grating period), the 561 nm (32 μm grating period), the 488 nm (32 μm grating period) and 405 nm (32 μm grating period) lasers, and filter set 3 (1850–553, Zeiss). 2D images were acquired using a sCMOS camera and processed using the ZEN software (2012, version 8.1.6.484, Zeiss). For channel alignment, a multicolored bead slide was imaged using the same image acquisition settings and used for the alignment of the different channels.

### Super-resolution Stimulated Emission Depletion (STED) microscopy

Time-gated STED imaging was carried out using a 100 x HC PL APO CS2 oil immersion objective (NA 1.4) on a Leica TCS SP8 STED 3x microscope running LAS X (Version 2.01.14392) acquisition software. GFP and Alexa Fluor 488 fluorescence were excited using the 488 nm line (8–10%) and mCherry fluorescence using the 561 nm output (10%) from a white light laser (WLL, operating at 70% of its nominal power). Fluorescence depletion, and therefore super-resolution, was accomplished using a 592 nm STED laser (1.5 W nominal power, 90% for GFP and 100% for Alexa Fluor 488). All 2048 × 2048 pixel single plane images were a acquired at a scan speed of 400 Hz in bidirectional scan mode. The pixel size of 14.20 nm^2^ was optimized for STED imaging. The fluorescence signal detected by a Hybrid Detector (HyD, Standard mode) after passing through an Acousto-Optical Beam Splitter (AOBS, detection range 495–585 nm for GFP alone, and 495–525 nm for Alexa Fluor 488 or GFP and 600–700 nm for mCherry when doing two-colour imaging). Time-gated detection was turned on to further improve the resolution in the STED images (0.3–6.0 ns for GFP and Alexa Fluor 488) or remove coverslip reflections in the confocal channel (0.5–8.0 ns for mCherry). The detector gain was adjusted so that no saturation occurred in the images.

### SIM Vaccinia Virus Analysis with VirusMapper

For each virion component modelled, 10,000–15,000 viral particles were extracted. For each model, seeds were created by averaging 3 to 6 individual, user selected, particles. For model generation, a similarity cut-off against the generated seeds was set between 95% and 99%, yielding a selection of ~1000 particles for model generation. In our investigations of vaccinia we found it necessary to distinguish between two orientations, frontal and sagittal, corresponding to the largest and second-largest face of the virus perpendicular to the optical axis respectively (Fig. 2b). Viruses were imaged on the coverslip in a random range of orientations, although we assumed cases where the virus was resting on its smallest face, in a transverse orientation, to be few. We distinguished between orientations in two ways. Firstly and most robustly, when imaging the WR L4-mCherry EGFP-F17 viruses the clear separation of the lateral bodies (F17 channel) reveals a sagittal orientation. We assumed those where only one lateral body was evident to be broadly in the frontal orientation. Secondly, in the case of the WR mCherry-A4 the core (A4 channel) was noticeably larger in the frontal orientation, consistent with the EM observations. Furthermore in some cases two maxima, arising due to the biconcave “peanut” shape of the core, were visible. This informed our choice of seed leading to separate frontal and sagittal models. The A4 and CellMask models involved this latter case to distinguish orientations and the remaining four models involved the former. We constructed the models as described in Supplementary Note 1. Seeds were chosen as the clearest representation of the frontal or sagittal orientation and the minimum similarity threshold was chosen to try to exclude particles of the wrong orientation.

### STED Vaccinia Virus Analysis with VirusMapper

For the STED models, around 5000 virus particles were extracted from series of images. Seeds and models were generated in the same way as for the SIM models, although with a similarity cutoff of between 92 and 95%, yielding around 500 particles per model.

### Vaccinia Virus Model Measurements and Cross-validation Analysis

To quantify the size of the core models (A4, L4 and DNA) we fitted a single Gaussian to the pixel values along the vertical and horizontal axes through the centre of the image and took the full width at half maximum (FWHM) as the length and width respectively. Similarly, we measured an estimate of the length and width of the lateral body model (F17). In the frontal and sagittal orientations the frontal length and width and the sagittal length are broadly the same. Viruses on the coverslip diverging from a frontal or sagittal orientation incorporated into the analysis contribute to the variation of these values. To quantify the size of the membrane models (A17 and CellMask) we fitted two Gaussians to the pixel values along the vertical and horizontal axes. Our estimates for the length and width are the combined width of these two Gaussians. That is, the distance between their centres plus half the FWHM of each. Borrowing from Szymborska *et al*.^6^ in order to estimate the precision of our model measurements we performed cross-validation analysis on the data. Each model was the average of at least 1000 particles. We then split these particles in random, non-overlapping subsets of 50 particles, averaged each of these subsets and made measurements for these averages, giving a range of values clustered around the model measurement. The precision was then calculated as the standard deviation of these measurements (Fig. 3b, and Supplementary Table 1). In Fig. 3 the precision for the difference measurements was obtained by adding in quadrature.

### Cell-based SR-SIM Sample Preparation

BSC40 cells were grown on coverslips (18 × 18 mm 1.5 H Zeiss). Purified virus was diluted in DMEM at a MOI of ~2, bound to cells for 60 minutes at room temperature and then samples were fixed in 4% formaldehyde for 20 minutes and washed 3 times with PBS. Cell membranes and nuclei were visualized with CellMask Green (ThermoFisher) and Hoechst (Life Technologies), respectively. Samples were imaged as described above for purified viruses. Z-stacks were taken over the full height of the cells at an interval of 100 nm. Maximum intensity projections were generated and were analysed with VirusMapper as described.

## Additional Information

**How to cite this article**: Gray, R. D. M. *et al*. VirusMapper: open-source nanoscale mapping of viral architecture through super-resolution microscopy. *Sci. Rep.* **6**, 29132; doi: 10.1038/srep29132 (2016).

## Supplementary Material

Supplementary Information
