# Supplementary material for: VirusMapper: open-source nanoscale mapping of viral architecture through super-resolution microscopy
Source: Sci Rep. 2016 Jul 4;6:29132. doi: 10.1038/srep29132 (PMC4931586; doi:10.1038/srep29132)
Supplement: Supplementary Information [file srep29132-s1.pdf]

Supplementary Information

VirusMapper: open-source nanoscale mapping of viral architecture through super-resolution microscopy

Robert D. M. Gray[*,1,2], Corina Beerli[*,1], Pedro Matos Pereira[1,3], Kathrin Scherer[1], Jerzy Samolej[1], Christopher Karl Ernst Bleck[4], Jason Mercer[||,1], Ricardo Henriques[||,1,3]

[1]MRC-Laboratory for Molecular Cell Biology. University College London, Gower Street, London, WC1E 6BT, United Kingdom

[2]Centre for Mathematics and Physics in Life Sciences and Experimental Biology (CoMPLEX), University College London, Gower Street, London, WC1E 6BT, United Kingdom

[3]Department of Cell and Developmental Biology, University College London, Gower Street, London, WC1E 6BT, United Kingdom

[4]Biozentrum, University of Basel, Klingelbergstrasse 50/70, CH-4056 Basel, Switzerland

* These authors contributed equally to this work

|| Correspondence: jason.mercer@ucl.ac.uk (J.M.); r.henriques@ucl.ac.uk (R.H.)

Supplementary Note 1

This section provides a detailed description of the functioning of the VirusMapper algorithms (Supplementary Fig. 1).

Extracting Viral Particles

Super-resolution images of large numbers of viruses bound to coverslips are segmented into individual particles using a peak detection algorithm based on QuickPALM [1]. The algorithm is initialized by finding the brightest pixel in each image and registering its location as a peak into a list. A binary mask is then generated demarking the area surrounding the detected peak as a region where no other peaks can be found on the proceeding iteration, thus avoiding peak overlap. Iteratively the algorithm repeats this process, finding the brightest pixel outside of the mask, thus capturing the local maximum of the image. For viral structures that do not feature a peak of intensity at its centre, we found that prior convolution with a Gaussian kernel of pre-defined size can generally form a peak of intensity close to the centre of the structure, which can then be used for peak detection. For each peak detected in the sequence of images, a square ROI of chosen radius around the peak is taken as the particle image and appended to an image list composed by ROIs featuring the segmented viral particles.

Seed Generation

Seed images need to be chosen to enable the template matching process to form an initial model. They are chosen from the set of segmented particles and may be clear representations of viruses in a certain orientation, or simply clearly imaged viruses. Multiple seed images may be combined into a single image by normalising and averaging. First, they are aligned with each other by fitting each image with a 2D elliptical Gaussian – we found that even for non-elliptical structures an elliptical Gaussian may provide a good approximation providing a major and minor axis of the shape. Images are then translated

and rotated to bring the centre of the fit into the centre of the image and the elliptical Gaussian major axis into the vertical. Application of a Gaussian blur to the image before fitting allows consistent alignment of most asymmetric shapes in this way.

Multiple fluorescence channels can also be incorporated. It may be that the orientation of the virus cannot be ascertained in one channel but it can in another. In this case, seeds for the two channels can be selected together, allowing differentiation between orientations to come from the second channel.

<u>Model Generation</u>

As a first step, individual viral particles are registered in location and rotation against the user generated seed. The displacement and rotation for each individual particle to provide maximum similarity with the seed is evaluated by calculating a normalized rotation and cross-correlation map (NRCCM).

In matching an image f, with pixel values $f(x,y)$, with a template t, with pixel values $t(x,y)$ the NRCCM is given by:

$$NRCCM(t_x, t_y, \theta) = \frac{1}{\sigma_f \sigma_t} \sum_{x,y} (f((x - t_x)\cos\theta, (y - t_y)\sin\theta) - \bar{f})(t(x,y) - \bar{t})$$

with $\bar{f}$ and $\bar{t}$ the mean pixel values and σ_f and σ_t the standard deviations. x and y run across the image. The peak intensity in (t_x, t_y, θ) space describes the translation and rotation required to maximize the similarity between the image and the template. We find this peak to subpixel resolution by locally fitting a 2D Gaussian and extracting the centroid.

We thus find the peak in the NRCCM and apply the corresponding translation and rotation to register each particle in the particle image list against the given seed. A first model is generated by a normalized projection average of all the elements of the particle image list weighted by their peak similarity given by the NRCCM. Thus the model m constructed from

N registered, normalised images f_i has pixel values where w_i is the peak value of the NRCCM for image f_i:

$$m(x,y) = \frac{1}{N}\sum_{i}^{N} w_i f_i(x,y)$$

In parallel to the weighted average, the weighted mean-square-error (MSE) of the model is also generated as:

$$MSE(m) = \frac{1}{N}\sum_{i}^{N}\sum_{x,y}\left(f_i(x,y) - m(x,y)\right)^2$$

By reiterating over the same steps of model generation, where the seed is replaced by the model generated, it becomes possible for models to evolve and converge into the most common structure represented in the particle image list while the MSE between models and viral particles decreases.

To visualise the MSE we also created mean-squared-error images to go along with each model (Supplementary Fig. 2). The pixel values for these images is given by:

$$MSE_{image}(m,x,y) = \frac{1}{N}\sum_{i}^{N}\left(f_i(x,y) - m(x,y)\right)^2$$

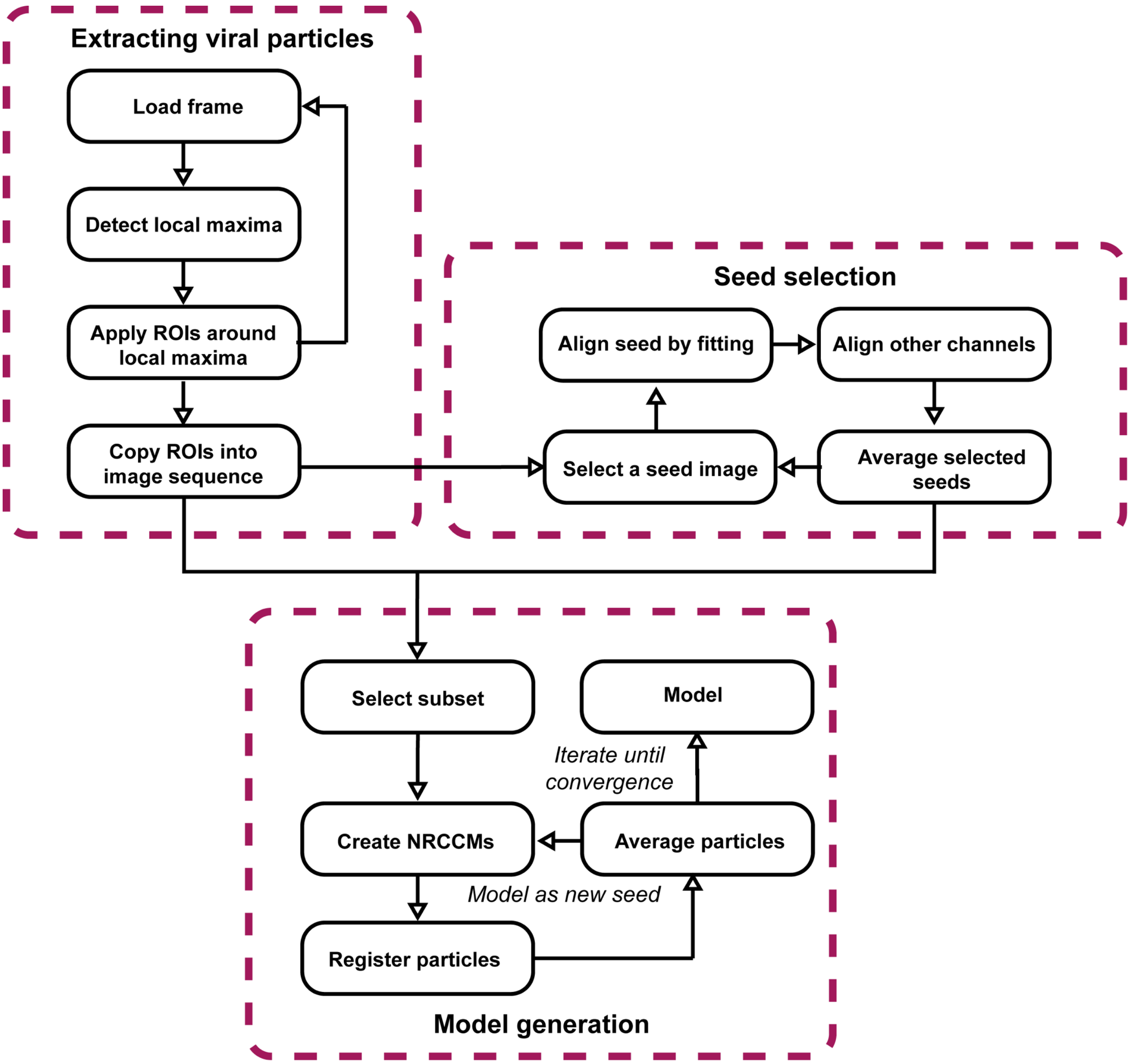

Supplementary Figure 1: VirusMapper software analysis framework. VirusMapper framework with its three main stages: extracting viral particles, seed selection and model generation.

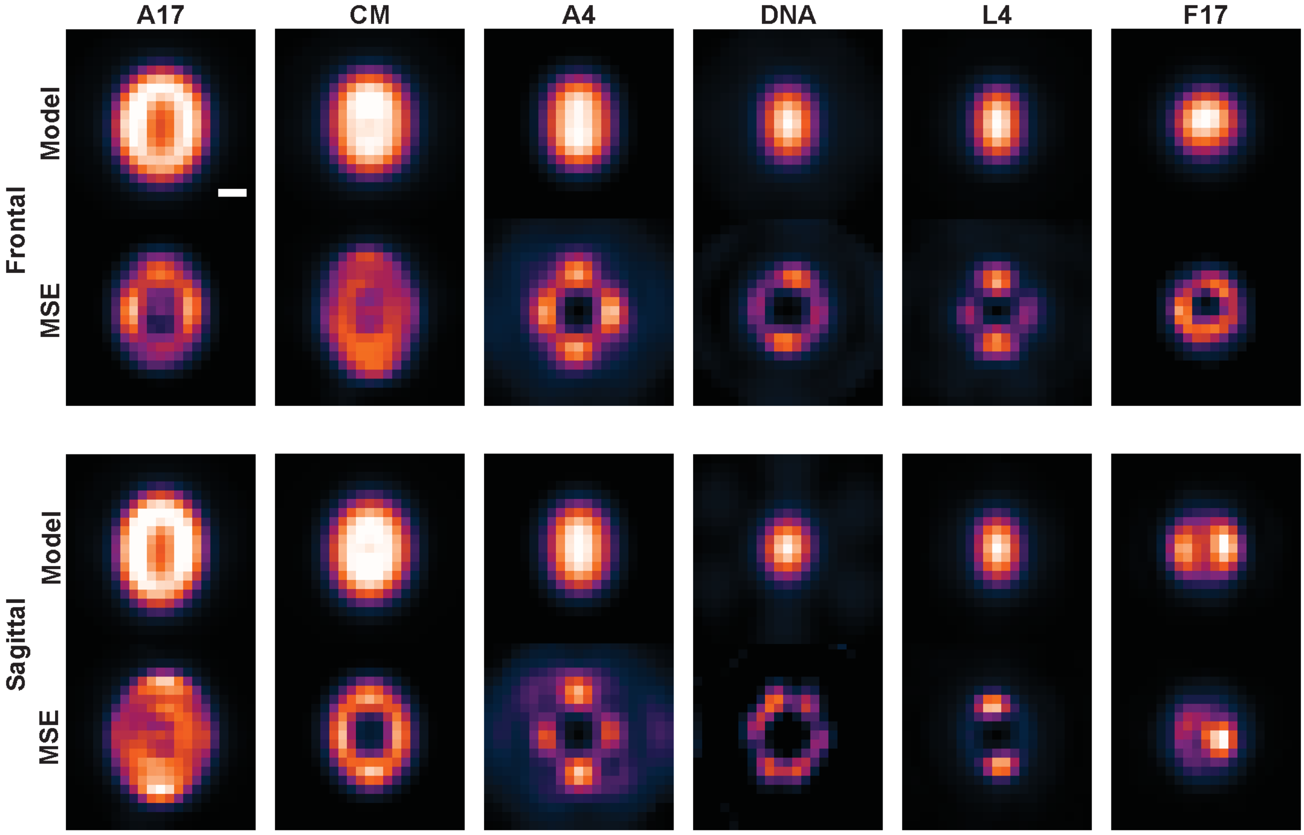

Supplementary Figure 2: Frontal and sagittal models of the six vaccinia components with their corresponding MSE image. This maps variability in the sets of images contributing to the model as described. Lookup tables for models and MSE are not the same; the values in the MSE images are much smaller and have been normalised for display.

Supplementary Table 1. Frontal and sagittal length and width measurements for the six vaccinia components modelled. Errors are the standard deviation of the cross-validation distribution for each. Each model was the average of at least 1000 particles. For statistical analysis random, non-overlapping subsets of 50 particles were averaged and measurements performed, giving a range of values clustered around the model measurement. The precision was then calculated as the standard deviation of these measurements.

Component	Frontal length	Frontal width	Sagittal length	Sagittal width
A17	425.8±4.1	346.1±2.9	429.5±4.8	333.7±2.2
CellMask (CM)	356.2±2.1	287.8±2.6	351.6±2.3	264.2±2.7
A4	281.2±2.2	210.6±1.5	269.9±1.6	203.1±1.9
DNA	263.2±2.3	202.0±2.8	261.1±2.3	189.6±3.7
L4	244.8±2.4	199.7±1.9	249.9±2.7	196.2±1.9
F17	215.6±2.3	212.9±1.9	203.7±6.7	152.0±11.0

References

1 Henriques, R. *et al.* QuickPALM: 3D real-time photoactivation nanoscopy image processing in ImageJ. *Nature methods* **7**, 339-340, doi:10.1038/nmeth0510-339 (2010).
